# Clinical Features and Patient-Reported Outcomes in a Cohort of Patients with Sjögren’s Disease

**DOI:** 10.3390/jcm14062027

**Published:** 2025-03-17

**Authors:** Rossana Izzetti, Chiara Cinquini, Giovanni Fulvio, Marco Nisi, Chiara Baldini, Antonio Barone

**Affiliations:** 1 Department of Surgical, Medical and Molecular Pathology and Critical Care Medicine, University of Pisa, 56126 Pisa, Italy; 2 Department of Clinical and Experimental Medicine, University of Pisa, 56126 Pisa, Italy

**Keywords:** diagnosis, oral, oral health, quality of life, Sjögren’s syndrome

## Abstract

**Background**: Sjögren’s disease (SD) is an autoimmune condition causing progressive salivary and lacrimal glands dysfunction following lymphocytic infiltration in the glandular tissue. SD patients are more prone to oral health impairment due to a reduction in salivary flow. This study evaluated the relationship between oral health, functional tests, and patient reported outcomes in a cohort of SD patients. **Methods**: Patients diagnosed with SD underwent complete dental examination, with the recording of the decayed–missing–filled teeth index (DMFT), probing pocket depth (PPD), full mouth bleeding score (FMBS), and full mouth plaque score (FMPS). Hyposalivation was assessed using the unstimulated whole saliva flow rate (UWS). Patients were administered the European League Against Rheumatism (EULAR) Sjögren’s Syndrome Patient Reported Index, EULAR Sjögren’s syndrome disease activity index, Oral Health Impact Profile-14 (OHIP-14), Patient Acceptable Symptom State questionnaires, and a visual analog scale for xerostomia (VASx). **Results**: Fifty patients in total were enrolled. Reduced UWS was associated with higher DMFT, FMBS, and FMPS. Significant correlation was observed for UWS with VASx and OHIP-14 (*p* < 0.05). **Conclusions**: Quality of life and oral health appear mildly impaired in SD patients as an effect of reduced salivary flow, with higher DMFT and tendency towards gingival inflammation and plaque accumulation.

## 1. Introduction

Sjögren’s disease (SD) is a chronic autoimmune disorder characterized by lymphocytic infiltration of the exocrine glands, resulting in dryness of mucosal surfaces, most notably the eyes and the mouth [1]. The diagnosis of SD is performed according to the criteria developed by the American College of Rheumatology/European League Against Rheumatism (ACR/EULAR), which include specific clinical and laboratory findings such as labial salivary gland biopsy results, the presence of Anti–Sjögren’s-Syndrome-Related Antigen A (anti-Ro/SSA) autoantibodies, ocular staining scores, Schirmer’s test, and unstimulated whole saliva flow rate (UWS) [2].

The reduction in salivary flow is among the primary manifestations of SD, contributing to the impairment of oral cavity health [3]. Saliva plays a crucial role in maintaining oral health through several protective mechanisms, due to the presence of enzymes, antimicrobial proteins, and ions that help to neutralize acids produced by bacteria, remineralize tooth enamel, and wash away food particles and microorganisms [4]. Consequently, the decrease in salivary flow predisposes patients to a spectrum of oral complications, ranging from dental caries and periodontal disease to mucosal infections and dysphagia [5]. A correlation between oral health and salivary flow has been hypothesized, demonstrating that patients with reduced salivary secretion experience significantly higher rates of dental caries and mucosal diseases compared to those with normal salivary flow [6]. Decreased salivary flow, or hyposalivation, can indeed result from various conditions and medications. Apart from SD, radiation therapy to the head and neck, diabetes, dehydration, and hormonal imbalances, particularly during menopause, can exacerbate xerostomia. Additionally, numerous medications are implicated in reducing salivary flow, including antihypertensives, antihistamines, antidepressants, antipsychotics, diuretics, and anticholinergics, which interfere with salivary gland function [7].

Xerostomia represents a major complaint among SD patients, contributing to an overall impairment in quality of life [8]. The constant dryness related to actual or perceived hyposalivation may lead to difficulties in speaking, chewing, and swallowing, which significantly impact daily activities and social interactions, further exacerbating the discomfort and malaise commonly experienced by SD patients [9]. Previous literature has extensively investigated the correlation between patient-reported outcomes, disease activity and symptom severity, with contrasting results. Objective outcome measures have been reported to be weakly correlated with patient-reported outcomes, as frequent discrepancies between functional test impairments and the subjective perception of symptoms can be observed [10]. However, higher scores on the Oral Health Impact Profile-14 (OHIP-14), which assesses the social and psychological impact of oral conditions, have been linked with increased disease activity and worse patient-reported outcomes, as measured using indices such as the EULAR Sjögren’s Syndrome Patient Reported Index (ESSPRI) and the EULAR Sjögren’s Syndrome Disease Activity Index (ESSDAI) [11].

The present study aimed to assess the oral health status of SD patients and its correlation with functional tests, alongside evaluating patient-reported outcomes to understand the broader impact of the disease on quality of life.

## 2. Materials and Methods

### 2.1. Study Design and Population

This was a single center, cross-sectional study on patients affected by SD referred to the Unit of Dentistry and Oral Surgery of the University Hospital of Pisa. The study protocol was approved by the Institutional Review Board of the University Hospital of Pisa (North-Western Tuscany Ethics Committee, approval no. 14540, 19 March 2019) and registered in a clinical trials database (clinicaltrials.gov, NCT06338735). All patients signed an informed written consent form to participate in the study. The study was conducted in compliance with the principles outlined in the Declaration of Helsinki. Consecutive patients with a confirmed diagnosis of SD according to the ACR/EULAR and who fulfilled the inclusion/exclusion criteria were enrolled in this study. The eligibility criteria are reported in Table 1.

### 2.2. Clinical Examination

Complete dental examination was carried out by a single calibrated examiner (intra examiner agreement > 0.9) with more than 10 years’ experience. The time from the last dental examination was recorded.

The decayed–missing–filled teeth index (DMFT), classified as low (0–4), moderate (5–13), and high (14 and above), was employed to assess overall dental health [12].

Probing pocket depth (PPD) was assessed by measuring the distance between the gingival margin and the deepest part of the periodontal pocket using a University of North Carolina-15 periodontal probe (UNC-15) with a calibrated pressure of 0.25 N. Measurements were taken at six sites per tooth: distobuccal, buccal, mesiobuccal, distolingual, lingual, and mesiolingual, and were rounded to the nearest millimeter [13].

Full mouth bleeding score (FMBS) was measured dichotomously after periodontal probing. The mean bleeding score was indicated as the percentage of sites categorized as positive for bleeding out of the total number of sites [14].

Full mouth plaque score (FMPS) was assessed as the presence or absence of plaque on each tooth surface. The presence of plaque was scored as 1, while the absence thereof was scored as 0, on six sites per tooth (distobuccal, buccal, mesiobuccal, distolingual, lingual, and mesiolingual). The mean plaque score was the percentage of sites classed as positive for the presence of plaque over the total number of sites [15].

UWS was registered by collecting saliva in the absence of any external or internal stimuli influencing salivary gland activity. The test was conducted in the morning at least one hour after eating, drinking, or oral hygiene practices. Saliva was collected by periodically spitting into a graduated container over a collection period of five minutes. The flow rate was calculated in milliliters per minute (mL/min). Normal UWS values range between 0.3 and 0.4 mL/min, while rates below 0.1 mL/min are indicative of hyposalivation, often associated with conditions such as xerostomia. Salivary flow was dichotomously categorized as <0.1 mL/min or ≥0.1 mL/min [16].

### 2.3. Questionnaire Administration

Four questionnaires were administered to the patients on the day of their dental examinations.

The OHIP-14 is a self-administered questionnaire designed to assess the social impact of oral disorders on an individual’s well-being [16]. The OHIP-14 focuses on capturing essential aspects of how oral health affects quality of life using 14 questions and covers seven domains reflecting different dimensions of oral health-related quality of life. These domains are functional limitation, physical pain, psychological discomfort, physical disability, psychological disability, social disability, and handicap [17]. A 5-point Likert scale (with the scores 0–4 corresponding to never, hardly ever, occasionally, fairly often, and very often) is used to assign scores to each item. The total OHIP-14 score can range from 0 to 56, with higher scores indicating a greater negative impact of oral health on quality of life. The scores can be interpreted as follows: 0–14 indicates a minimal impact, 15–28 indicates a moderate impact, 29–42 indicates a high impact, and 43–56 indicates a severe impact.

The ESSPRI is designed to measure symptoms in SD patients, by focusing on the three key domains of pain, fatigue, and dryness [18]. The questionnaire asks patients to rate their experiences over the past two weeks in the three domains, with each item being rated on a 0–10 numeric rating scale (NRS). The mean scores obtained from the three domains result in an overall score ranging from 0 to 10. Higher scores indicate more severe symptoms and a greater impact on the patient’s quality of life.

The ESSDAI is a comprehensive tool used to assess the systemic manifestations of the disease across multiple organ systems, thereby providing an overall measure of disease activity [19]. The questionnaire includes 12 domains (constitutional, lymphadenopathy, glandular, articular, cutaneous, pulmonary, renal, muscular, peripheral nervous system, central nervous system, hematological, and biological), with each representing a different organ system or aspect of the disease. For each domain, the level of disease activity is scored on a scale from 0 to 3 or 0 to 4, depending on the domain. The total score typically falls within the range of 0 to around 123.

The patient-acceptable symptom state (PASS) is a patient-reported outcome measure designed to assess whether patients consider their current symptom state to be satisfactory by answering the question “Considering all the different ways SD is affecting you, if you were to stay in this state for the next few months, do you consider your current state satisfactory?” [20,21]. It focuses on capturing the patient’s overall perception of their symptom burden and its impact on their quality of life. The PASS provides a straightforward assessment of patient satisfaction with their symptom control and overall well-being. A “Yes” response indicates that the patient feels their symptoms are manageable and not significantly impacting their quality of life and generally corresponds to an ESSPRI score of <5. A “No” response suggests that the patient is experiencing a symptom burden that is affecting their daily functioning and well-being, with an ESSPRI score of ≥5. The PASS is used to measure treatment outcomes and patient satisfaction, providing a valuable complement to clinical and laboratory assessments. It offers insights into the patient’s subjective experience, which is crucial for comprehensive disease management.

The visual analogue scale for xerostomia (VASx) assesses the severity of dry mouth through 8 items (difficulty when talking, difficulty when swallowing, quantity of saliva, level of mouth, throat, lips and tongue dryness, and level of thirst) [22]. The VASx score is expressed as a mean of the values of the 8 items, providing a quantitative measure of xerostomia severity, which can be used to track changes over time, evaluate the effectiveness of treatments, and understand the impact of dry mouth on the patient’s quality of life.

### 2.4. Sample Size Calculation

The sample size calculation was performed on the basis of the study performed by Pedersen et al. [23], with an expected r or Rs value of 0.4, and aimed to obtain a level of significance of 5%. Accordingly, at least 47 subjects were needed to reach a power of 80%; a final sample of 50 participants was selected to compensate for potential dropouts.

### 2.5. Statistical Analysis

All analyses were carried out using Jamovi software (The jamovi project, Version 2.5, https://www.jamovi.org, accessed on 9 May 2024). A Shapiro–Wilk test was employed to verify the normal distribution of data. Descriptive statistics were employed to summarize the clinical and analytical characteristics of the patients, presenting categorical variables as counts and percentages, and quantitative variables as means ± standard deviations (normal distribution) or medians and confidence interval (CI) (non-normal distribution). According to the distribution, Spearman or Pearson correlation indexes with Bonferroni-corrected significance levels were employed to analyze the relationships between the UWS and the quality-of-life questionnaires administered. The significance level was set at α = 0.05.

## 3. Results

### 3.1. Population Characteristics

Fifty patients (46 females and 4 males) were enrolled. The median time from disease diagnosis was 13 months [CI 11.91; 14.02] (Table 2). All patients who enrolled completed the study.

### 3.2. Oral Health Parameters

All the patients had visited the dental office in the previous year, with a median time since the last examination of 7 months [CI 6.91; 7.68]. A total of 1278 teeth were analyzed, with a median of 25 teeth per patient [CI 23.63; 27.02]. Among the participants, two patients wore partial removable dentures, and 21 patients had at least two fixed prosthetic crowns. The analysis revealed 36 decayed teeth, 322 missing teeth, and 247 filled teeth, resulting in a median DMFT score of 10 [CI 8.25; 12.6]. The median PPD was 4 mm [CI 3.65; 5.19], with 123 sites in 23 patients measuring ≥ 5 mm. The mean FMBS was 39%, and the mean FMPS was 42%. The median UWS was 0.24 mL/min [CI 0.18; 0.33]. In Table 3, the correlation between oral parameters and UWS is reported.

UWS was significantly negatively correlated with DMFT (*p* < 0.001), indicating that higher salivary flow rates were associated with lower DMFT scores. It also showed strong negative correlations with both FMBS and FMPS (*p* < 0.001), suggesting that increased salivary flow was linked to better oral hygiene and less bleeding. DMFT was moderately positively correlated with FMPS (*p* < 0.001), implying that higher DMFT scores were associated with higher plaque scores. PPD showed a moderate positive correlation with FMBS (*p* < 0.05), indicating that greater pocket depths were associated with more bleeding sites.

### 3.3. Questionnaire Scores and Disease Activity

The median score for the ESSDAI was 11 [CI 5.77; 15.56], reflecting a moderate disease activity. OHIP-14 had a median score of 24 [CI 23.64; 26.48), suggesting a moderate impact of oral health on quality of life. The ESSPRI median score was 6.67 [CI 5.61; 6.81], indicating a moderate level of symptom severity reported by patients. The VASx had a median score of 7 [CI 6.28; 7.37], reflecting a relatively high average rating of overall experience or symptom severity, with some degree of variability in responses (Table 4).

There was a moderate positive correlation between OHIP-14 and ESSPRI (Spearman’s Rho = 0.368, *p* < 0.001). This indicated that higher OHIP-14 scores were associated with higher ESSPRI scores, suggesting that more severe symptoms reported by patients were linked to increased oral health impacts. There was a strong positive correlation between ESSPRI and VASx (Spearman’s Rho = 0.657, *p*< 0.001). This suggested that higher ESSPRI scores, indicating greater patient-reported symptom severity, were closely related to higher VASx scores, as both measures reflected a similar overall assessment of symptom severity and patient experience. A weak positive correlation existed between OHIP-14 and VASx (Spearman’s Rho = 0.239, *p* < 0.001), with higher OHIP-14 scores being associated with more severe xerostomia.

OHIP-14 correlations with ESSDAI (Spearman’s Rho = −0.007, *p* = 0.915) and PASS (Spearman’s Rho = 0.022, *p* = 0.737) were very weak, with near-zero values indicating no meaningful relationship, and were not statistically significant. Similarly, ESSPRI showed very weak non-significant correlations with ESSDAI (Spearman’s Rho = −0.026, *p* = 0.693) and PASS (Spearman’s Rho = 0.048, *p* = 0.464), suggesting minimal to no relationship with these variables. The VASx also showed weak, non-significant correlations with PASS (Spearman’s Rho = −0.042, *p* = 0.511) and ESSDAI (Spearman’s Rho = −0.006, *p* = 0.926), both indicating weak negative correlations that are not statistically significant.

### 3.4. Correlation Between Questionnaires and Oral Parameters

The correlation scores between oral parameters and questionnaires are reported in Table 5. The correlation matrix revealed significant relationships between UWS and several questionnaire measures. Specifically, UWS showed a significant negative correlation with OHIP-14 (Spearman’s Rho = 0.257, *p* = 0.021), indicating that reduced salivary flow rates were associated with impaired oral health-related quality of life. Additionally, UWS was negatively correlated with both ESSPRI (Spearman’s Rho = −0.311, *p* = 0.007) and VASx (Spearman’s Rho = −0.291, *p* = 0.012), suggesting that higher salivary flow rates were linked to lower patient-reported symptom severity and better overall experience, respectively. In contrast, the correlations between UWS and other parameters, including ESSDAI, PASS, DMFT, FMBS, FMPS, and PPD, were not statistically significant. These results highlighted the importance of salivary flow in influencing patients’ perceived oral health and overall symptom burden. No significant correlations were observed between the other oral parameters (DMFT, FMBS, FMPS, and PPD) and the questionnaire measures, indicating a lack of strong relationships in these cases.

## 4. Discussion

The present results indicate a clear association between a reduced UWS and adverse dental outcomes, such as a higher DMFT index, increased FMBS, and higher FMPS. These results are consistent with previous studies, which have shown that reduced saliva production leads to an increased prevalence of dental caries, periodontal disease, and oral infections. Interestingly, decreased salivary flow affected quality of life, as confirmed by the negative correlation with OHIP-14, ESSPRI, and VASx. These results suggest that reduced UWS impacts these measures of health and well-being, and subsequently oral health, symptom severity, and overall patients’ experience of the disease.

The strong negative correlations between UWS and dental parameters such as FMBS and FMPS underscore the critical role of saliva in maintaining oral health. Saliva not only facilitates the mechanical cleansing of the oral cavity but also provides essential enzymes and proteins that inhibit bacterial growth and promote tissue repair [24]. In patients with SD, the lack of sufficient saliva creates a conducive environment for the proliferation of pathogenic bacteria, leading to higher plaque accumulation and gingival inflammation [25]. This was evident in our cohort, where patients with UWS < 0.1 mL/min exhibited significantly higher FMBS and FMPS scores compared to those with higher salivary flow rates.

Interestingly, while an increase in DMFT, FMBS, and FMPS was noted in the presence of impaired UWS, no correlation was observed in terms of risk of developing periodontitis. The impact of SD on oral health has been previously extensively discussed in the literature. Molania et al. [26] reported no statistically significant differences in periodontal indices or DMFT between SD patients and controls. However, a meta-analysis by Maarse et al. [27] evaluating the occurrence of periodontal disease in SD patients showed that, although periodontal parameters were generally higher in SD patients, these differences were not statistically significant, except for the DMFT index, which was higher in SD patients. The authors hypothesized that the reduced salivary secretion in SD patients could be a contributing factor to increased plaque accumulation and slightly worse gingival health, although these differences were not clinically significant [27]. Another systematic review [28] investigating the potential link between SD and an increased risk of periodontal disease suggested a trend towards poorer periodontal health in SD patients, but the results were not statistically robust, largely due to the inconsistent quality and methodology of the included studies. The literature therefore conveys the fact that, while an overall higher gingival inflammation and plaque accumulation can be observed in SD patients [29], no higher occurrence of periodontitis can be found in patients with SD as compared to the general population [30]. Indeed, we can speculate that the decrease in salivary flow may lead to an increased plaque accumulation and subsequent gingival inflammation, although the hyposalivation may favor the shift towards carious pathogens rather than periodontal ones. The literature has previously indicated that, with SD, the microbial ecosystem becomes specialized in amino acid fermentation, resulting in a higher salivary pH and the increased production of bacterial deaminases and proteases, which can trigger inflammation and increase the likelihood of shifting towards dysbiosis [31]. Therefore, microbiota present in SD is more susceptible to transformation into cariogenic biofilm, thus contributing to an increased risk of dental caries [32]. Our findings appear to be consistent with the existing literature, highlighting the need for more careful monitoring of oral health in SD patients to prevent the onset of oral health complications, which could negatively impact their overall quality of life.

Patient-reported outcomes are crucial for understanding the subjective experience of disease and its impact on quality of life. The significant correlations between VASx, OHIP-14, and UWS highlight the profound effect of dry mouth on patients’ daily lives. Our findings are consistent with the previous literature, as Serrano et al. [11] also reported a significant positive correlation between VASx and OHIP-14 scores, as well as a significant negative correlation between UWS and OHIP-14 scores. In our sample, the mean OHIP-14 score was slightly higher (25.06) but still comparable to previous studies by Serrano et al. [11] (23.13), Stewart et al. [33] (23.7) and Amaral et al. [34] (21.2). A recent systematic review with a meta-analysis [30] found a mean OHIP-14 score of 15.3 in studies comparing SD and sicca patients. However, the limited number of eligible studies and the differences in sample dimensions were reported as potential confounding factors.

Despite hyposalivation being a key symptom in SD, its correlation with self-reported quality of life has been rarely studied. Previous studies [35,36] reported weak and non-significant correlations between UWS and OHIP-14 scores. However, our results highlighted a significant negative correlation between UWS and OHIP-14 scores. When comparing UWSs of <0.1 mL/min and ≥0.1 mL/min, significant differences in OHIP-14 scores were observed.

Overall, the results support the use of the OHIP-14 questionnaire as a reliable tool for assessing the impact of xerostomia and hyposalivation on quality of life in SD patients. This aligns with the findings by Baker et al. [37], corroborating the fact that OHIP-14 is effective in assessing quality of life in relation to xerostomia in rheumatic patients. Additionally, the significant positive correlation between UWS and VASx suggests that the severity of hyposalivation and the degree of xerostomia significantly influences patients’ quality of life. The positive correlations between OHIP-14 and both ESSPRI and ESSDAI scores indicate that patients with more active disease and severe symptoms perceive a greater impact on their oral health-related quality of life. This relationship highlights the interconnection of systemic disease activity and oral health outcomes in SD; indeed, patients experiencing higher levels of pain, fatigue, and mouth dryness are more likely to report difficulties in performing daily activities, social interactions, and overall well-being. This multidimensional impact necessitates a comprehensive approach to address both systemic and oral manifestations of the disease [38].

The findings of this study have several important implications for clinical practice. First, regular dental assessments should be an integral part of the treatment plan for patients with SD. The early detection and management of dental caries, periodontal disease, and other oral health issues can prevent more severe complications and improve patients’ quality of life [39]. Moreover, providing prosthetic rehabilitations of missing teeth to SD patients may lead to an improvement in quality of life [40,41]. Dental professionals should be aware of the unique challenges faced by SD patients and adopt preventive strategies tailored to their needs. A multidisciplinary approach involving both dentists and rheumatologists can ensure that patients receive comprehensive care addressing both the systemic and oral aspects of their condition.

While this study provides valuable insights into the relationship between hyposalivation, dental health, and patient-reported outcomes in SD, there are some limitations requiring further investigation. The demographic heterogeneity within the sample group might have introduced variability in the results, thus limiting the generalizability of the findings. The cross-sectional design of the study also limits the ability to draw causal inferences. The absence of longitudinal follow-up prevents an assessment of long-term effects, and measurement errors or variations in data collection could impact the consistency of the results. Finally, the sample may not be fully representative of the broader population, and the lack of a control group may hinder the generalizability of the results. The DMFT index was employed to assess dental status; however, the lack of further radiographic investigations may have led to an underestimation of the amount of caries. Additionally, follow-ups of incipient carious lesions were not performed. Finally, a re-assessment of SD patients to evaluate whether oral conditions tend to deteriorate with SD progression was not performed.

## 5. Conclusions

Hyposalivation and disease activity significantly impact both objective dental parameters and subjective patient-reported outcomes in individuals with SD. Higher DMFT scores and an increased tendency towards gingival inflammation and plaque accumulation were observed in SD patients, highlighting the need for dedicated oral treatment plans to improve the oral health and the daily lives of affected individuals. Future research should continue to explore innovative solutions and therapeutic options to enhance the management of this complex autoimmune condition.

## Figures and Tables

**Table 1:** Eligibility criteria for participation in the study.

Inclusion Criteria	Exclusion Criteria
Males or females of age > 18 years	Diagnosis of secondary Sjögren’s disease
Sjögren’s disease diagnosis in the absence of other rheumatologic diseases	History of treatment with sialagogues
Xerostomia not treated pharmacologically	Pregnancy/breastfeeding or systemic conditions hindering participation to the study
Ability and willingness to give informed consent	Unwillingness to participate in the study.

**Table 2:** Baseline sample characteristics.

Sample Characteristics	
Age (years)	53 [CI 49.05; 56.08]
Gender	46 females, 4 males
Presence of SD (months)	13 [CI 11.91; 14.02]
Positivity to ACR/EULAR criteria	
Oral symptoms (oral dryness)	45 (90%)
Ocular symptoms (ocular dryness)	46 (92%)
UWS < 0.1 mL/min	23 (46%)
Ocular signs	35 (70%)
Histology of minor salivary glands (Focus Score ≥ 1)	34 (68%)
Positive Anti-Ro/SSA	28 (56%)
Disease activity	
Parotid inflammation	5 (10%)
Musculoskeletal involvement	12 (24%)
Skin involvement	9 (18%)
Pulmonary involvement	3 (6%)
Renal involvement	1 (2%)
Central nervous system involvement	2 (4%)
Peripheral nervous system involvement	0 (0%)
Hematological involvement	4 (8%)
Gastrointestinal involvement	7 (14%)

Abbreviations: ACR: American College of Rheumatology; Anti Ro/SSA: Anti–Sjögren’s-Syndrome-Related Antigen A Autoantibodies; CI: Confidence Interval; EULAR: European League Against Rheumatism; SD: Sjögren’s disease; UWS: unstimulated whole saliva flow rate.

**Table 3:** Correlation matrix of oral parameters.

Correlation Matrix—Spearman’s Rho (*p*-Value)					
	UWS	DMFT	PPD	FMBS	FMPS
UWS	—				
DMFT	−0.382 (<0.001)	—			
PPD	−0.127 (0.282)	0.142 (0.243)	—		
FMBS	−0.419 (<0.001)	0.105 (0.361)	0.299 (<0.05)	—	
FMPS	−0.356 (<0.001)	0.379 (<0.001)	0.117 (0.318)	0.267 (<0.05)	—

Abbreviations: DMFT: decayed–missing–filled teeth; FMBS: full mouth bleeding score; FMPS: full mouth plaque score; PPD: probing pocket depth; UWS: unstimulated whole saliva flow rate.

**Table 4:** Correlation matrix of questionnaires scores.

Correlation Matrix—Spearman’s Rho (*p*-Value)					
	OHIP-14	ESSPRI	ESSDAI	PASS	VASx
OHIP-14	—				
ESSPRI	0.368 (<0.001)	—			
ESSDAI	−0.007 (0.915)	−0.026 (0.693)	—		
PASS	0.022 (0.737)	0.048 (0.464)	0.015 (0.818)	—	
VASx	0.239 (<0.001)	0.657 (<0.001)	−0.006 (0.926)	−0.042 (0.511)	—

Abbreviations: ESSDAI: EULAR Sjögren’s Syndrome Disease Activity Index; ESSPRI: European League Against Rheumatism (EULAR) Sjogren’s Syndrome Patient Reported Index; OHIP-14: Oral Health Impact Profile-14; PASS: patient acceptable symptom state; VASx: visual analog scale for xerostomia.

**Table 5:** Correlation matrix of questionnaires scores versus oral parameters.

Correlation Matrix—Spearman’s Rho (*p*-Value)					
	OHIP-14	ESSPRI	ESSDAI	PASS	VASx
UWS	−0.159 (<0.05)	−0.311 (<0.05)	−0.035 (0.803)	0.102 (0.403)	−0.291 (<0.05)
DMFT	−0.055 (0.675)	−0.077 (0.569)	0.112 (0.362)	−0.039 (0.732)	0.049 (0.702)
PPD	0.142 (0.251)	0.108 (0.376)	−0.058 (0.659)	−0.019 (0.761)	0.094 (0.465)
FMBS	−0.144 (0.245)	−0.126 (0.317)	0.065 (0.622)	−0.091 (0.478)	−0.128 (0.310)
FMPS	−0.135 (0.281)	−0.109 (0.373)	0.081 (0.549)	−0.104 (0.395)	−0.117 (0.351)

Abbreviations: DMFT: decayed–missing–filled teeth; ESSDAI: EULAR Sjögren’s Syndrome Disease Activity Index; ESSPRI: European League Against Rheumatism (EULAR) Sjogren’s Syndrome Patient Reported Index; FMBS: full mouth bleeding score; FMPS: full mouth plaque score; OHIP-14: Oral Health Impact Profile-14; PASS: patient acceptable symptom state; PPD: probing pocket depth; UWS: unstimulated whole saliva flow rate; VASx: visual analog scale for xerostomia.

## Data Availability

Data are contained within the article.
